# Microarray expression analysis of meiosis and microsporogenesis in hexaploid bread wheat

**DOI:** 10.1186/1471-2164-7-267

**Published:** 2006-10-19

**Authors:** Wayne Crismani, Ute Baumann, Tim Sutton, Neil Shirley, Tracie Webster, German Spangenberg, Peter Langridge, Jason A Able

**Affiliations:** 1 Molecular Plant Breeding Cooperative Research Centre, School of Agriculture, Food and Wine, The University of Adelaide, Waite Campus, PMB1, Glen Osmond, South Australia, 5064, Australia; 2 Australian Centre for Plant Functional Genomics, School of Agriculture, Food and Wine, The University of Adelaide, Waite Campus, PMB1, Glen Osmond, South Australia, 5064, Australia; 3 Molecular Plant Breeding Cooperative Research Centre and Australian Centre for Plant Functional Genomics, Victorian AgriBiosciences Centre, La Trobe University, 1 Park Drive, Bundoora, Victoria, 3083, Australia

## Abstract

**Background:**

Our understanding of the mechanisms that govern the cellular process of meiosis is limited in higher plants with polyploid genomes. Bread wheat is an allohexaploid that behaves as a diploid during meiosis. Chromosome pairing is restricted to homologous chromosomes despite the presence of homoeologues in the nucleus. The importance of wheat as a crop and the extensive use of wild wheat relatives in breeding programs has prompted many years of cytogenetic and genetic research to develop an understanding of the control of chromosome pairing and recombination. The rapid advance of biochemical and molecular information on meiosis in model organisms such as yeast provides new opportunities to investigate the molecular basis of chromosome pairing control in wheat. However, building the link between the model and wheat requires points of data contact.

**Results:**

We report here a large-scale transcriptomics study using the Affymetrix wheat GeneChip^® ^aimed at providing this link between wheat and model systems and at identifying early meiotic genes. Analysis of the microarray data identified 1,350 transcripts temporally-regulated during the early stages of meiosis. Expression profiles with annotated transcript functions including chromatin condensation, synaptonemal complex formation, recombination and fertility were identified. From the 1,350 transcripts, 30 displayed at least an eight-fold expression change between and including pre-meiosis and telophase II, with more than 50% of these having no similarities to known sequences in NCBI and TIGR databases.

**Conclusion:**

This resource is now available to support research into the molecular basis of pairing and recombination control in the complex polyploid, wheat.

## Background

Bread wheat (*Triticum aestivum *L.) is an allohexaploid (2n = 6x = 42) containing three related genomes: A, B and D, with significant conservation of gene order between the chromosomes of the respective genomes. Early mapping studies including those conducted by Chao *et al*. [1] using RFLPs to map markers on chromosome group 7 confirmed strong conservation of gene order between chromosomes of each genome. Indeed, this conservation is also seen when comparing wheat with other grasses. While several papers have been published reporting synteny between the Poaceae genomes at the macro level [2,3], microsynteny appears to be perturbed frequently [4,5]. The current comparative genomics model reported by Devos [3] segregates nine major monocotyledonous genomes into 25 'rice linkage blocks' and provides a valuable base for gene discovery and genome structural analysis in grass species leading out from the sequenced rice genome.

While comparative genomics has enabled the identification of putative orthologues in many cereals, there are processes or characteristics that are either species specific, such as quality, or for which wheat provides a good model, such as the control of chromosome pairing during meiosis. During meiosis, DNA undergoes a round of replication, chromatin is condensed and the genetic content is halved twice after two rounds of chromosome segregation and cell division. Classical cytogenetic techniques suggest that the fundamental events and processes of meiosis are conserved amongst higher eukaryotes. The production of viable gametes at the conclusion of meiosis requires completion of several critical processes during prophase I that include chromosome pairing, synapsis and recombination.

During meiosis in bread wheat, strict regulation of homologous chromosome pairing is maintained, despite the presence of homoeologous chromosomes. This maintains the stability of the complex wheat genome but is also a limitation in utilizing the extensive variation available in wild relatives of wheat. Several hundred wild grasses can be crossed to wheat but introgression of desirable alleles from these species is dependent upon the induction of pairing and recombination with the wheat chromosomes. Several genes, particularly *Ph1*, are known to block or enhance homoeologous pairing; with removal of this locus shown to induce novel pairing and recombinational behaviour. An understanding and ability to modify the mechanisms that control pairing, synaptonemal complex formation and recombination would be of great benefit to crop improvement programs. Such information would also enhance our understanding of complex polyploidy genomes and factors influencing fertility.

While functional studies of individual genes have been useful for advancing our knowledge of meiosis, very few resources are available to examine whole genome expression changes during meiotic development. A broad resource base will be important to help identify conserved processes between organisms, and conversely, highlight processes that have diverged. Microarray studies have provided valuable data on meiosis and related cellular processes in several species. An early study in *Saccharomyces cerevisiae *used a microarray chip with 97% of the then known budding yeast genes [6], which increased the number of known meiotically-regulated genes from approximately 150 to over 1,000. A more recent experiment with budding yeast compared the meiotic transcriptomes of two strains with different sporulation efficiencies and identified approximately 1,600 meiotically-regulated genes in each strain with a 60% overlap between the two strains [7]. While these two examples are yeast specific, other model organisms have also been used to study meiosis with microarray technology, including fruit flies (*Drosophila melanogaster*), mice (*Mus musculus*), nematodes (*Caenorhabiditis elegans*) and rats (*Rattus rattus*) [8-11].

The current study provides information that links to the transcript profiling work conducted in yeast and animal systems and to the extensive cytological and genetic data for wheat to provide a view of meiosis in a complex polyploid and one of the world's most important agricultural crops. Using the Affymetrix GeneChip^® ^Wheat Genome Array, which contains 61,127 probe sets (55,052 transcripts) (Affymetrix), 1,350 transcripts were found to be expressed across a sub-staged meiotic time series of whole wheat anthers. Four hundred and sixty seven have no detectable similarities to entries in sequence databases. While there have been extensive studies on the duration and timing of meiosis in bread wheat [12-15] and also on chromatin condensation and chromosome pairing [16-18], the transcript *in silico *research presented here advances our limited understanding of meiosis at the molecular level and complements the previous *in planta *studies cited.

## Results and Discussion

The basic aim of the work outlined here was to develop a view of gene expression over the various stages of meiosis in wheat. The resulting data set helps define the wheat meiome (meiotic transcriptome) and will provide a key resource for work aimed at explaining the control of meiosis and chromosome pairing in a complex polyploid relative to diploid plant, animal and yeast systems.

### Comparison of tissue expression profiles

There are two key components of the experimental system. First, whole anthers were used for preparing the RNAs. The meiocytes themselves form a major proportion of the tissues in the anthers but RNA from other cell types, notably epidermal cells and tapetum, will be included in the extracts and will contribute to the results. Therefore, the data represents gene expression from a number of different cell types with meiocytes as the major constituent.

The second component is the Affymetrix wheat GeneChip^®^. This is a 'discovery' chip and is not representative of the complete wheat genome nor does it allow differentiation between genes expressed from the three wheat genomes. Further, many of the sequences used to design the chip were not of high quality. Therefore, data generated from this chip must be carefully checked and key results confirmed using alternative expression analysis methods. In addition, it is important to note that at the time the wheat GeneChip^® ^was designed, wheat meiotic floret cDNA and cDNA from anthers undergoing meiosis represented approximately 6% of the total representations available in the wheat EST database. Therefore, the wheat GeneChip^® ^is assumed to provide a good representation of meiotic expressed genes.

**Figure 1:**
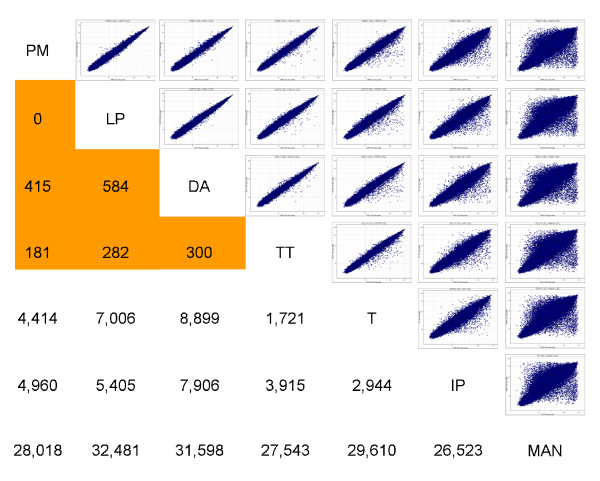
**Scatter plot and statistical analysis comparing all seven stages investigated with the wheat Affymetrix GeneChip^® ^to one another**. PM = pre-meiosis; LP = leptotene to pachytene; DA = diplotene to anaphase I; TT = telophase I to telophase II; T = tetrads; IP = immature pollen; MAN = mature anthers. All values in the figure represent the number of transcripts with corrected p-values ≤ 0.05 from t-tests performed between the two tissues of interest. The orange shading represents the pooled stages from where the 1,350 non-redundant meiotically-regulated transcripts were identified.

Scatter plots illustrated the similarity of replicates, and the broad relationship of the expression profiles from pre-meiosis through to the tetrad stage (Figure 1). However, marked differences were apparent in the expression profiles of both immature pollen and mature anthers when compared to each other and the meiotic-specific sub-stages; pre-meiosis through to tetrads. Given that both of these tissues and the regulation of gene expression during pollen development is complex, this level of variance is not surprising. It has been shown in several plants including tomato (*Lycopersicon esculentum*) and maize (*Zea mays*) that up to 70% of plant genes are expressed post-meiotically [19,20]. A study of the barley transcriptome also found that anthers were the most complex of tissues [21]. The results presented here reflect these findings with 67% of transcripts expressed in tetrads, immature pollen and/or mature anthers (RMA average signal intensity > 5).

### Gene filtration, identification and analysis of meiotically-regulated transcripts

The Affymetrix wheat GeneChip^® ^contained 61,127 probe sets, which was reduced to 55,000 upon the removal of control, reporter, duplicate and 'rogue' probe sets (see Methods – Microarray analysis). The majority of the available annotations for the probe sets are based on homologies to genes from organisms other than wheat. This was due to the lack of sequence information, characterised genes and their protein products for wheat.

**Figure 2:**
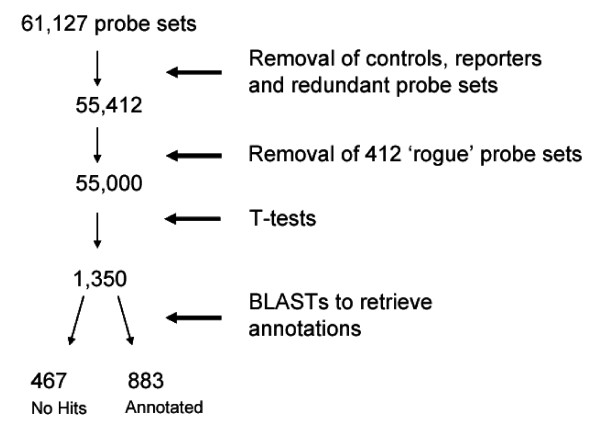
**Filtration process used for Affymetrix wheat GeneChip^® ^data**. The controls and 'rogue' probe sets were removed from the data prior to filtration. Meiotically-regulated transcripts were then identified using t-tests, with NCBI database annotations retrieved for the remaining transcripts where available (*E*-value < e^-10^).

T-tests between expression levels at different stages of meiosis were used to identify genes showing regulation of expression during meiosis. The comparisons made were as follows: pre-meiosis (PM) with leptotene-pachytene (LP); PM with diplotene-anaphase I (DA); PM with telophase I-telophase II (TT); LP with DA; LP with TT and DA with TT (p values = 0.05) (Figure 1). This resulted in the classification of transcripts from 1,350 non-redundant genes as being meiotically-regulated of which 467 had no annotation (Figure 2). A similar strategy was used by Schlecht and colleagues [10] with a rat data set to examine both meiosis and gametogenesis. The rat analysis resulted in the identification of 1,268 diverse meiotic transcripts from a total of 11,955.

**Figure 3:**
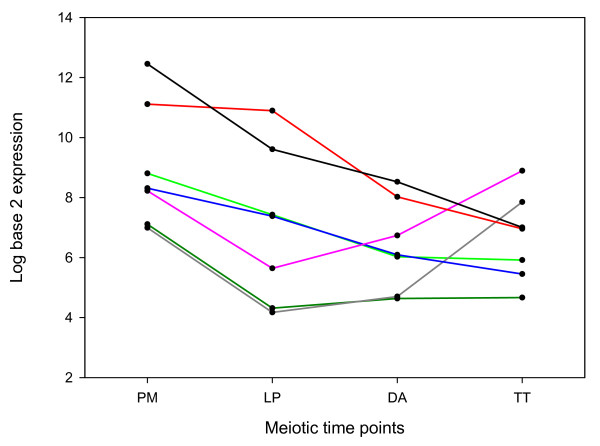
**Transcripts with at least an eight-fold change in expression level between and including pre-meiosis to telophase II**. Black = TaAffx.9800.1.S1_at; red = TaAffx.60258.2.S1_s_at; light green = Ta.10020.1.S1_at; blue = Ta.6922.1.S1_at; pink = TaAffx.38162.1.S1_at; dark green = Ta.16669.1.S1_x_at; and grey = Ta.6831.1.S1_at. PM, LP, DA and TT are as described in Figure 1.

When compared to the 55,000 relevant probe sets on the current wheat GeneChip^®^, the 1,350 meiotically-regulated transcripts represent only 2.45%. Thirty prominent profiles within the 1,350 meiotically-regulated transcripts showed at least an eight-fold expression change between combinations of PM, LP, DA and TT. Of these 30 transcripts, no similarities to any database entries currently on record (August 2006) could be found for 16. Consequently, these represent a source of new and potentially wheat meiosis-specific transcripts. Seven of the 30 transcripts significantly change in level of expression during the first three meiotic time points investigated in this study (Figure 3). It is during these stages where events including chromosome pairing, synapsis and homologous recombination occur. The temporal expression of the genes encoding these transcripts at the time points where homologous recombination occurs make them particularly interesting targets for further work aimed at investigating homologous pairing and recombination.

The mature anthers (MAN) accounted for the majority of variability detected (73%) between transcript profiles across the developmental stages. This is consistent with work on pollen development in *Tradescantia *and maize. Pollen germination and pollen tube growth are two processes that carry a significant metabolic cost, since it involves storage of mRNA and other organic molecules [22]. Previous studies of pollen development have typically used inhibitors of transcription and translation to show that mRNAs are produced during pollen maturation and stored for use during pollen germination [for reviews see [23,24]]. Further, many of the protein products of these genes are already present in mature pollen prior to germination [25-27].

Transcript expression during pollen maturation is generally defined as either early or late, with early transcripts present in microspores subsequently down regulated before pollen maturation, while late transcripts are produced and accumulated after pollen mitosis I [reviewed in [28]]. *In situ *studies from tomato and maize suggest that many of the late genes are the product of the vegetative cell [29,30]. Studies in both *Tradescantia *and maize have revealed that about 10% of mRNAs stored in mature pollen grains are pollen-specific [31,32].

Over the seven stages investigated in this study, 38,242 transcripts were differentially expressed in mature anthers when compared to the first four stages of the meiotic time course (p ≤ 0.05 between PM v MAN, LP v MAN, DA v MAN and TT v MAN). Similarly, when immature pollen (IP) was compared to the four meiotic stages, considerable variation was still detected with 9,385 transcripts showing differential expression.

### Expression analysis in the developing wheat anther

**Figure 4:**
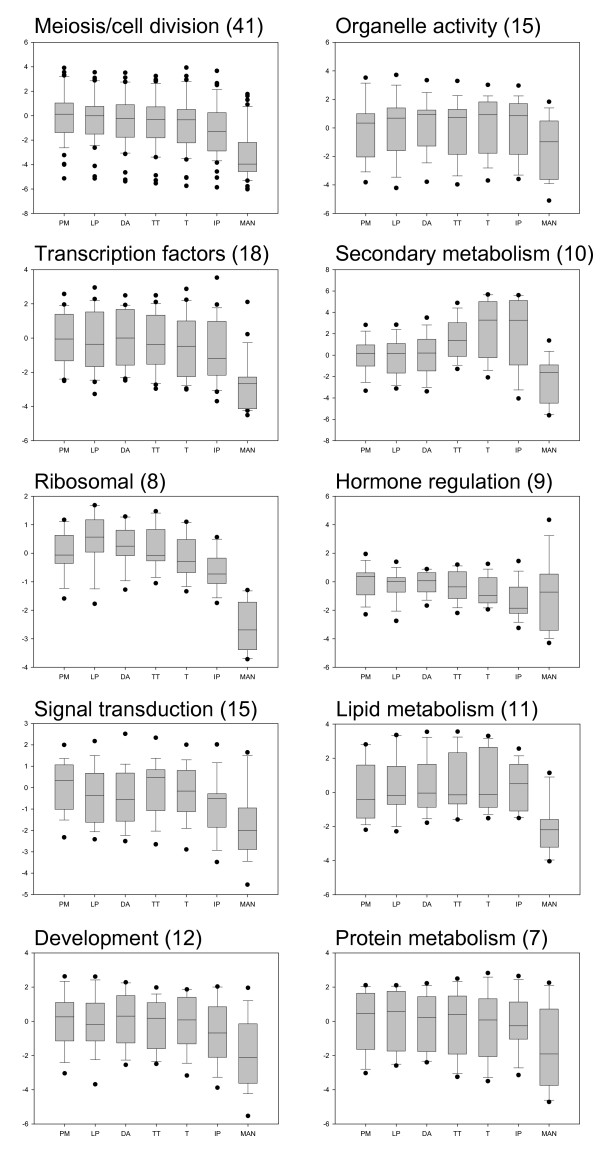
**Functional classifications and expression analysis from the developing wheat anther**. Transcripts were pooled into categories based upon their annotations and having satisfied the following criteria: an *E*-value < e^-30^, a sequence length > 250 bp and their cross-hybridizing status (based on the Affymetrix designation). The data for each functional category is represented as log base 2, RMA normalized values that were centered on the average of the pre-meiosis (PM) value. Representations (n) assigned to each functional category are indicated in brackets.

The complexity of the anther tissues used in this study meant that interpretation of the data required a consideration of developmental changes that were occurring in anther cells other than just the meiocytes. To obtain a view of gene expression changes that may be indicative of the meiotic process and also general developmental shifts, ten biological categories of potential significance were used to group members of the 1,350 diverse transcripts found to show variable expression (see Methods – Microarray analysis). These groups covered genes likely to be involved in tissue growth and differentiation such as those related to hormone regulation, signal transduction and development, as well genes involved in metabolic process. 146 probe sets fell into one of ten categories (Figure 4).

During the first five meiotic stages used in this study, the majority of the categories showed no significant increase or decrease in the level of expression, indicating a degree of transcriptional stability in the anthers over these stages of development. However, there were more pronounced effects when comparing the mature anther stage across all 10 functional categories. In general, all 10 categories showed decreased levels of expression during this stage of development when compared to the other tissues investigated. This trend is particularly evident in the meiosis/cell division and ribosomal categories, inferring that the mRNA of these candidates is down-regulated in mature anthers. While not as significant, the transcription factors, organelle activity, signal transduction, lipid metabolism, development and protein metabolism categories also displayed this downward trend as the anther matured. This decline is occurring as the pollen matures and reflects the shutting down of cell division, expansion and differentiation.

### Meiotic clusters

#### Hierarchical clustering

**Figure 5:**
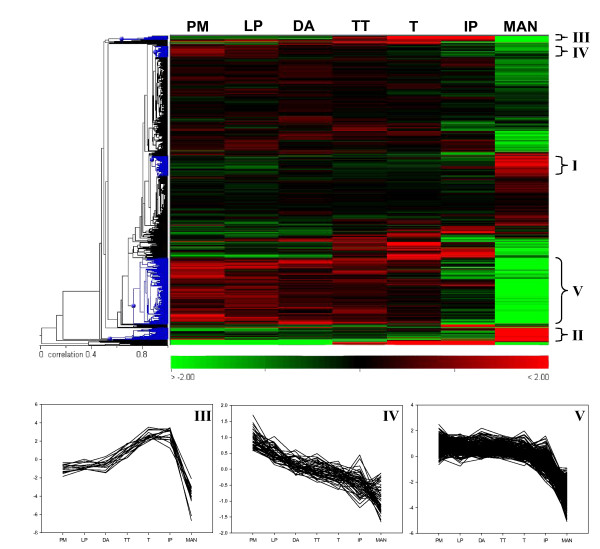
**Hierarchical clustering of 1,350 meiotically-regulated transcripts**. The expression profiles of 1,350 transcripts (rows) were grouped across seven staged anthers (columns); pre-meiosis (PM), leptotene to pachytene (LP), diplotene to anaphase I (DA), telophase I to telophase II (TT), tetrads (T), immature pollen (IP) and mature anthers (MAN) (averages shown). Clusters of interest (described in text) are highlighted in blue and labelled I and II (pollen clusters); III, IV and V (meiotic clusters). The Y-axis for cluster groups III, IV and V is centered, log base 2, RMA normalized values.

Hierarchical clustering of the 1,350 meiotically-regulated transcripts was performed to identify common gene expression patterns (Figure 5). This revealed two clusters with 88 and 50 transcripts (indicated by I and II on Figure 5) that were expressed at low levels throughout pre-meiosis to immature pollen, only to then rise sharply at the onset of mature anther development (fold changes of 12.91 and 3.12, respectively). Within these two clusters, there were a number of transcripts present with known annotations to genes that have roles in flower development. One such candidate is the GRAB2 protein [33], which contains a NAM (no apical meristem) domain. NAM domains are part of the NAC domain family [34], which have been shown to have roles in cell division and cell expansion in floral organs, as well as determining the positions of meristems and primordia [35]. Another identified transcript, *NEC1*, has been reported to have a role in the opening of anthers in petunia (*Petunia hybrida*) [36].

In addition to these flowering candidates, two *PIP1 *aquaporin transcripts that have previously been isolated from wheat were identified. One could speculate that the up-regulation of aquaporins during pollen maturation helps prevent damage to reproductive structures. Previous work suggests that the products of this gene family help prevent chilling injury [37]. Although other annotated candidates were identified from these two clusters, approximately 53% of the transcripts showed no similarity to any known genes or proteins in the public databases (August 2006). An investigation of the roles of these unknown transcripts is likely to be useful in clarifying the developmental process involved in pollen maturation in wheat and other cereals. In particular, these genes are expressed shortly before full pollen maturity and dehiscence. As the anthers used to prepare this RNA were still green, at this stage there will be genes likely to be involved in preparing the pollen for release. This is consistent with the putative role for the *PIP *genes in desiccation protection. Investigation of other genes in this group may provide valuable targets for general stress or desiccation tolerance.

Although the meiotic stages examined in this study could have been more tightly defined (for example, leptotene could have been separated from pachytene), the timing and collection of such specific events during wheat meiosis would not have guaranteed producing improved resolution. While Chu and colleagues [6] and Primig *et al*. [7] divided their meiotic profiles into seven different categories, these experiments used yeast, where synchronization and identification of specific individual meiotic stages is simple. The meiotic time series used here is significantly more detailed than those examined in other plant systems. Meiosis I and II in bread wheat anthers is completed within 24 hours, with prophase I (which includes leptotene, zygotene, pachytene, diplotene and diakinesis) taking 17 hours [14]. In contrast to other grasses, this is one of the most rapid to complete meiosis with rye (*Secale cereale*) taking 51 hours and barley (*Hordeum vulgare*) taking 39 hours [15]. This is partially explained by Bennett and Smith [14] who showed that in wheat, rye and *Triticale *the duration of meiosis decreases as the ploidy level increases.

Besides cluster groups I and II previously described, at least three clusters (indicated by III, IV and V on Figure 5) appeared to be meiotically-regulated, suggesting that meiotic genes undergo subtle changes in expression, rather than sharp increased or decreased bursts of expression. Possible reasons accounting for these observations include; 1) there may not be a large number of genes exclusively involved in meiosis, irrespective of the number of genes within the organism's genome; 2) meiosis classifications are largely based on cytological observations and may not reflect what is occurring at the transcript level, and 3) the 'dilution effect' of other tissues present in wheat anthers. It should be noted that during meiosis, chromatin is heavily condensed and this may pose limitations for the transcriptional machinery of the cell. Consequently, the expectation would be for gene expression to be restricted to genes essential to the meiotic process. Transcripts required for other functions, such as general cell metabolism, would most effectively be provided prior to the onset of meiotic condensation.

Of the meiotically-regulated clusters identified, one cluster contained only 15 transcripts, while the other two contained 48 and 287 transcripts (indicated on Figure 5 by III, IV and V respectively). The cluster of 15 transcripts (III) showed enhanced expression during the latter stages of meiosis I. Consequently, these genes may be involved in the latter stages of meiosis or the early stages of pollen maturation. While 33% of these transcripts showed no similarity to any known sequences in the public databases (August 2006), one transcript was similar to a gene encoding a male fertility protein from both maize (Accession Number: NP_912416) and wheat (Accession Number: AAV70496) suggesting that this transcript has a role in pollen maturation as opposed to meiosis. When examining the other two cluster groups (indicated by IV and V on Figure 5), many transcripts were identified that have already been reported to have roles in processes that occur during early meiosis. Not only were transcripts that had significant similarities to cell cycle proteins, kinesin-related proteins and chromomethylases identified but candidates implicated in chromosome pairing, synapsis and recombination were also represented.

**Figure 6:**
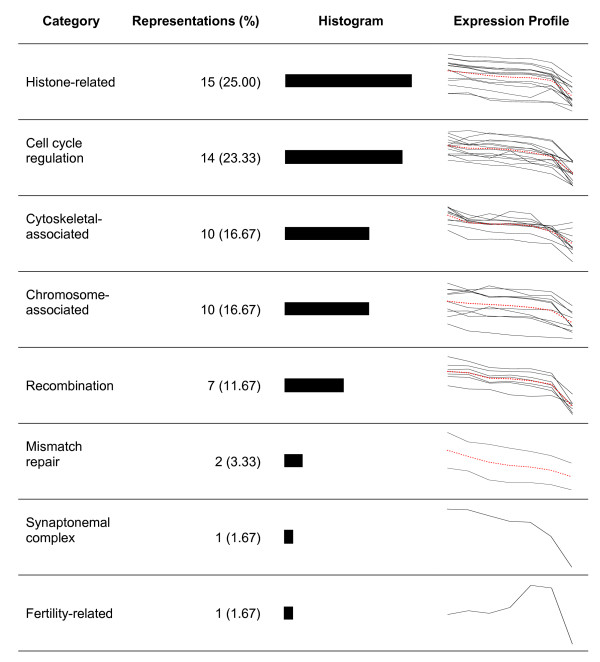
**Functional classifications for the 60 meiosis and/or cell division transcripts identified from Table 1**. These transcripts were classified into functional categories based on the annotation obtained. The histogram represents the absolute values for each category, while the expression profiles for each of the candidates within each of the categories has also been shown. The red dotted line in the expression profile graphs represents the overall trend line for that category.

To gain insight into the functional roles of genes identified in these three clusters a similar strategy to that of Iguchi and colleagues [38] was used, whereby transcripts were assigned to a biological category based on annotated functions. In cluster groups III, IV and V, approximately 40% of the 350 transcripts (pooled value from the three clusters) were either functionally not annotated or novel (with no database matches returned) (Table 1). While the classifications of the 350 transcripts ranged from functional categories as diverse as abiotic stress-related and secondary metabolism, a reasonable proportion (17%) represented unambiguous meiotic and/or cell division candidates (Table 1). These transcripts were assigned to a specific meiotic and/or cell division category based on their annotation (Figure 6). Transcripts showing significant database matches to histones, cell cycle and cytoskeletal candidates isolated from other organisms, as well as chromosome-associated, recombination and mismatch repair genes were all identified.

### Transcripts involved in early meiosis

The key aim of the experiments described here was to identify genes that may be involved in controlling the early stages of meiosis. Therefore we were particularly interested in transcripts that showed expression only in the early meiotic stages. These were identified based on transcripts with an RMA normalized value > 6 for either PM or LP and < 5 for all stages thereafter. Interestingly only four transcripts were identified, of which three (TaAffx.11970.3.S1_s_at, TaAffx.29005.1.S1_at and TaAffx.86700.1.S1_at) showed no significant similarity to sequences in the NCBI and TIGR databases. The remaining transcript (Ta.400.2.A1_at) returned a significant hit of 6e^-59 ^to a putative condensin complex subunit from rice (BLASTx). The condensin complex has been reported to have varying roles during both mitosis and meiosis, including chromosome segregation and spindle assembly. These genes are clearly prime candidates for further study since they might have a role in coordinating the early meiotic event of recombination.

From the 1,350 putative meiotic transcripts identified through gene filtration, many appear to be orthologous to genes from other organisms that are known to be involved in meiosis. Specifically, there are representatives of genes involved in processes such as synapsis, *TaASY1 *(an orthologue of *AtASY1*, *Arabidopsis *asynapsis 1) (Boden *et al*., unpublished data). Furthermore, there are several mismatch repair genes including wheat MutS homologue 2 (*TaMSH2*), wheat MutS homologue 6 (*TaMSH6*), and the previously characterised wheat MutS homologue 7 (*TaMSH7*) [39]. Other putative meiotic transcripts identified within the microarray data include candidates for recombination, such as radiation sensitive *RAD51B *and *RAD51C*, disrupted meiotic cDNA (*DMC1*) and the rice replication protein A1 (RPA). Osakabe and colleagues [40] and Bleuyard and colleagues [41] have previously characterised *Arabidopsis *RAD51B (*AtRAD51B*) and found that it is important for double stranded DNA break repair in somatic cells. RAD51C on the other hand, is necessary for homologous recombination in meiosis and mitosis [41-43]. From previously published research, it is evident that RPA is essential for various aspects of DNA metabolism in eukaryotes [for review see [44]]. In rice and *Arabidopsis *there are two different types of RPA [45] with inactivation of the *AtRPA70a *resulting in lethality, thus suggesting a role in DNA replication. However, even though the phenotype appeared normal under standard growth conditions, disruption of *AtRPA70b *resulted in hypersensitivity to mutagens such as UV-B and methyl methanesulfonate. This implied a role in DNA repair processes [45].

Many of the transcripts with significant similarities to previously characterised meiotic genes (some of which have been described above) displayed analogous patterns within our data set. Therefore it appears that the wheat genes identified here are indeed orthologues of the meiotic genes identified in other organisms. Information on transcript abundance can help identify previously uncharacterized genes that may have roles in homologous recombination. Such transcripts would show co-regulation with known meiotic genes from within this meiotic data set. This assumption is supported by the close similarities in expression profiles between two related mismatch repair genes, *TaMSH7 *[39] and *AtMLH1 *[46] (correlation coefficient of 0.997).

**Figure 7:**
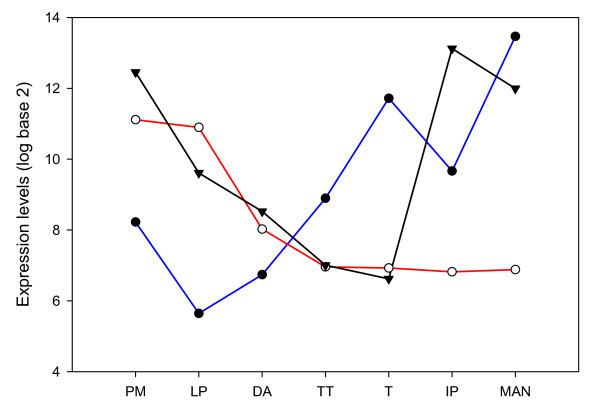
**Three novel candidates with significant expression variability across the seven stages investigated**. TaAffx.38162.1.S1_at (blue), TaAffx.9800.1.S1_at (black) and TaAffx.60258.2.S1_s_at (red) are highlighted. TaAffx.38162.1.S1_at shows more than a 64-fold increase in expression from LP to T, while TaAffx.9800.1.S1_at drops approximately 64-fold from PM to T. TaAffx.60258.2.S1_s_at is also down-regulated from PM to T approximately 16-fold. PM, LP, DA, TT, T, IP and MAN are as described in Figure 1.

In addition to the 'known' meiotic genes described above, the wheat data set contains many meiotic transcripts that do not show significant similarity to sequences in the NCBI and TIGR databases (*E*-value < e^-10^). Three of these transcripts display radically different levels of expression across the meiotic time course investigated (Figure 7). In particular, the transcript corresponding to the Affymetrix probe set TaAffx.38162.1.S1_at displays an extreme expression pattern in the microarray data. This expression profile was confirmed in the Q-PCR analysis. With more than a four-fold drop from PM to LP, and then a rapid 64-fold increase in expression between LP to T, this gene might encode a repressor of an event occurring within the leptotene to pachytene stages such as chromosome pairing, synapsis or recombination. Significantly, there does not appear to be a transcript within the entire data set that shares a similar expression profile to probe set TaAffx.38162.1.S1_at. Consequently, TaAffx.38162.1.S1_at is a worthy candidate for further work to elucidate its role during bread wheat meiosis.

### Q-PCR confirmation of microarray data

Q-PCR was conducted in order to validate representative microarray results and examine the correlation between the two expression profiling platforms. A total of 15 primer sets were designed to amplify fragments representing probe sets on the Affymetrix GeneChip^® ^(Table 2). Transcripts were selected based on their meiotic expression profiles and similarity to known meiotic genes (where there was an expression profile to support conservation of function).

**Figure 8:**
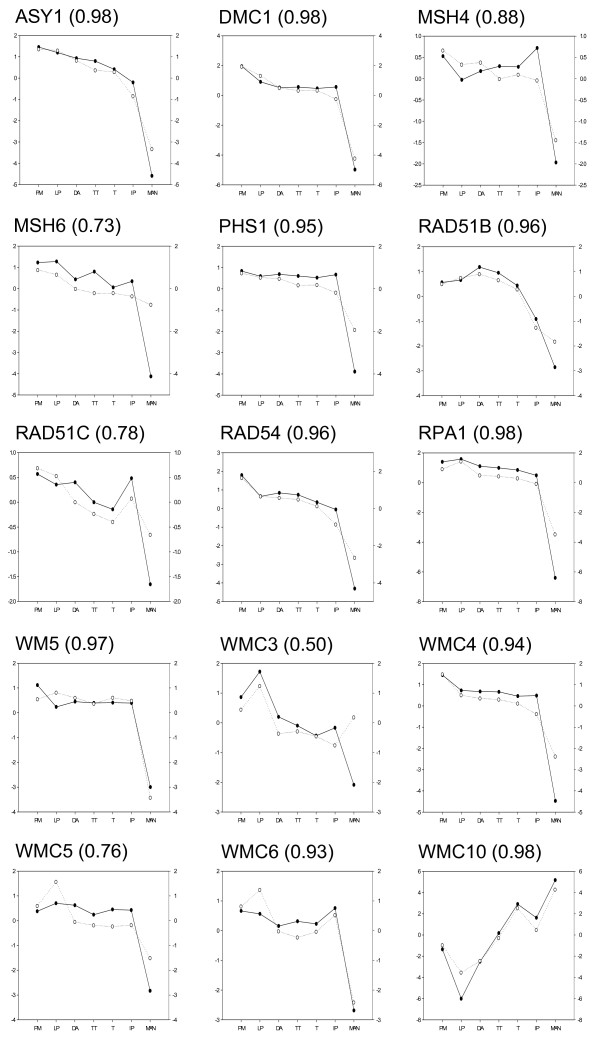
**Correlation of the microarray profile with the Q-PCR data from the corresponding transcript**. Lines with the open circles represent microarray data, while lines with solid circles represent the Q-PCR data. The data for each transcript from the microarray is log base 2, RMA normalized and centered about its average across the seven stages, while the data for the Q-PCR is log base 2, normalized mRNA copies/μL and centered. Correlation values for each of the transcripts investigated using both technology platforms are indicated.

The microarray and Q-PCR profiles were closely related. Correlation coefficients ranged from R = 0.73 to R = 0.98, with the exception of one outlier (R = 0.50) (Figure 8). The median correlation coefficient for the microarray/Q-PCR data set was R = 0.95. Overall expression patterns were similar, confirming a high degree of reproducibility between the two platforms. These results parallel those of Honys and Twell [47] and Shary and colleagues [48] who showed similar levels of correlation with reverse transcription PCR and northern blots, respectively.

A pairwise comparison between the Q-PCR results highlighted co-expression between many of the transcripts (Table 3) with a significant number of these transcripts having a correlation of over 0.9. These findings suggest that there are a discrete number of transcription factors that control meiotic genes and hence the regulation of meiotic progression in bread wheat, as has been suggested for yeast [49,50]. In order to further our understanding of meiotic regulation and determine whether any of these candidates are co-regulated, a yeast one-hybrid approach could be employed using the promoter regions of the candidates.

## Conclusion

The transcript profiling information generated in this study was designed to help decipher the bread wheat meiome as an important step to manipulate pairing and recombination in plant breeding programs. Over 1,300 transcripts showed meiotic regulation over the tissue series used in this study, with 467 novel transcripts identified. Through hierarchical clustering 60 meiosis and/or cell division candidates were identified as meiotically-regulated. Histone-related, chromosome-associated, recombination and fertility candidates were among those identified in this group. Through comparison with similar research in simpler eukaryotes, it has been possible to draw parallels with the more complex genome of hexaploid bread wheat. A select number of novel candidates in addition to known meiotic genes will now form the basis for further research. The key objective of the study outlined here, has been to provide a resource for meiosis research that can link the strong cytogenetic, genetic and practical research base of wheat to the detailed biochemical and molecular information now emerging from model organisms such as yeast.

## Methods

### Tissue isolation and RNA extraction

Wild-type wheat (*Triticum aestivum *L. cv. Chinese Spring) was grown in a temperature-controlled glass house at 23°C (day) and 15°C (night) with a 14 hour photoperiod. Anthers were collected and dissected using a Leica MZ6 dissecting microscope. Anther squashes were prepared in order to determine the stage of meiosis. Squashed anther preparations were viewed using a Leica DM1000 compound microscope.

The seven stages collected were pre-meiosis (PM), leptotene to pachytene (LP), diplotene to anaphase I (DA), telophase I to telophase II (TT), tetrads (T), immature pollen (IP) and mature anthers (MAN). Immediately after determining the stage, the remaining two anthers from the floret were placed into liquid nitrogen. After collecting at least 25 staged anthers for each time point from several biological samples, anthers from the respective stages were pooled. Leaf material used in this study was collected from glasshouse-grown plants (six weeks) and total RNA isolated using Trizol^® ^(Gibco BRL, Australia) according to the manufacturer's protocol.

### Microarray processing

aRNA from the seven stages were amplified and labelled from one microgram of total RNA with two rounds of amplification. The GeneChip^® ^Two-Cycle cDNA Synthesis Kit (Affymetrix, CA, USA) was used to produce reverse transcribed double-stranded cDNA containing a T7 promoter, which was *in vitro *transcribed using the Ambion Megascript T7 amplification kit (Ambion, TX, USA). This unlabelled aRNA was subsequently reverse transcribed to double stranded cDNA using the GeneChip^® ^Two-Cycle cDNA Synthesis Kit and biotin labelled aRNA was then produced using the GeneChip^® ^IVT Labelling Kit (Affymetrix, CA, USA). Twenty micrograms of aRNA from each of the seven samples were fragmented for hybridization to each microarray. In total three technical replicates were conducted for each of the seven stages examined. Affymetrix GeneChip^® ^Wheat Genome Arrays were used for all samples. The arrays were hybridized and processed according to the manufacturer's specifications.

### Microarray analysis

Normalization of the microarray data was conducted using RMA. The software package Acuity 4 (Molecular Devices, CA, USA) was then used to analyze the microarray data. Microarray expression ratios were presented as log-transformed base 2. Gene annotations were obtained through batch BLASTs (BLASTx; *E*-value < e^-10^) using the Affymetrix probe set sequences in tandem with sourcing annotations directly from the Affymetrix website. In total, 412 'rogue' probe sets were discarded from the analysis due to technical problems during the microarray processing.

All p-values referred to in the text are corrected p-values that were obtained using student's t-tests assuming equal variances and using the Benjamini-Hochberg method [51]. These p-values were used to determine whether transcripts were temporally-regulated during meiosis.

Suitable probe sets from the 1,350 transcripts used for expression analysis in the developing wheat anther were selected based on several criteria. Probe sets designated by Affymetrix not to cross-hybridize outside of their gene family were selected if they had a consensus sequence > 250 bp and had a BLASTx match with an *E*-value < e^-30^. The 633 transcripts that remained were then assigned to functional categories of interest based on literature searches of the BLASTx matches. The average of the pre-meiosis (PM) values for each respective functional category was then subtracted from every data point. The line graphs and box and whisker plots presented here were produced using Sigma Plot (Version 9) (Systat Software, IL, USA).

Hierarchical clustering analysis focussed on transcript expression patterns rather than absolute values and the triplicate data over the seven stages was averaged. The average of every row (that is, every transcript) was then subtracted from every signal intensity in the gene expression matrix. The rows were then clustered using a Euclidean squared similarity metric and the average linkage method. Acuity 4.0 (Molecular Devices, CA, USA) was also used to apply principal component analysis to the data set.

### Microarray data set

The microarray data set has been deposited in the Gene Expression Omnibus (GEO) database (Accession: GSE6027).

### cDNA synthesis and Quantitative Real-Time PCR

Seven independent cDNA synthesis reactions were made for the staged anthers (PM, LP, DA, TT, T, IP and MAN) using the same RNA that was hybridized to the wheat GeneChip^®^. RNA (1 μg) was used as template for the cDNA synthesis reaction with Superscript III RNAse H^- ^Reverse Transcriptase (Invitrogen, Australia) according to the manufacturer's instruction.

For each gene investigated using Q-PCR, a dilution series covering seven orders of magnitude was prepared from 10^9 ^copies/μL stock solution as detailed in Burton and colleagues [52]. Three replicates of each of the seven standard concentrations were included with every Q-PCR experiment together with a minimum of three no template controls. Q-PCR experiments were assembled by a liquid handling robot, a CAS-1200 robot (Corbett Robotics, Australia). Three replicate PCRs for each of the cDNAs were included in every run.

Two μL of the cDNA solution or the diluted standard or water was used in a reaction containing 5 μL of IQ SYBR Green PCR reagent (Bio-Rad Laboratories, California, USA), 1.2 μL each of the forward and reverse primers at 4 μM, 0.3 μL of 10X SYBR Green in water and 0.3 μL of water. The total volume of this PCR was 10 μL.

Reactions were performed in a RG 3000 Rotor-Gene Real Time Thermal Cycler (Corbett Research, Australia) as follows; 3 minutes at 95°C followed by 45 cycles of 1 second at 95°C, 1 second at 55°C, 30 seconds at 72°C and 15 seconds at the optimal acquisition temperature described in Table 2. A melt curve was obtained from the product at the end of the amplification by heating from 70°C to 99°C. Using the Rotor-Gene V6 software (Corbett Research, Australia) the optimal cycle threshold (CT) was determined from the dilution series, with the raw expression data derived. The mean expression level and standard deviation for each set of three replicates for each cDNA was calculated.

### Quantitative Real-Time PCR normalisation

The normalisation strategy by Burton and colleagues [52] was used in this time course experiment. Four control genes were assessed (*actin*, *GAPdH*, *EFA *and *cyclophilin*). The three best control genes from this set were selected, with normalisation factors calculated using the geNorm program [53].

While these genes have been used in other studies the consistency of expression of the best three control genes in this case was found to be poor. A measure of consistency was obtained by examining the M value [53], where a high M value indicates that a control gene has a very disparate expression with respect to other control genes. The highest M value from the best three genes in these experiments was approximately 1.7. Based on other time course experiments conducted, we anticipated M values of approximately 1.0. To address this concern, further control genes were tested until an acceptable M value was obtained. Two additional genes were selected from the microarray data (Ta.9657.1.S1_at and Ta.28350.1.S1_a_at) in addition to the four 'standard' Q-PCR control genes mentioned above.

Selection criteria imposed for identifying the additional control transcripts were based on a similar strategy to Czechowski and colleagues [54]. Additionally, the signal intensities were greater than five (log base 2) in all 21 arrays with the triplicate data for the transcripts having a standard deviation of less than 0.1. Probes sets that Affymetrix denoted as being likely to cross hybridize were not selected. After re-calculating the normalization factors using geNorm, the highest M value from the three best control genes was reduced to 0.68 and the raw expression values for the genes of interest in each cDNA were divided by the normalization factor for that cDNA to produce the normalized expression data.

## Authors' contributions

WC conducted the research, analyzed the data and drafted the manuscript. UB and TS designed the research, analyzed the data and drafted the manuscript. NS, TW and GS contributed analytical tools and analyzed the data. PL designed the research and drafted the manuscript. JAA designed and conducted the research, analyzed the data and drafted the manuscript. All authors read and approved the final manuscript.

## Figures and Tables

**Table 1:** Functional classifications for 350 meiotically-regulated transcripts.

**Category**	**Representations (%)**
Meiosis/cell division	60 (17.1)
Cellular metabolism	24 (6.86)
Transcription factors and DNA binding	18 (5.14)
Ribosomal	15 (4.29)
Biotic stress-related	12 (3.43)
Membrane transport	12 (3.43)
Signal transduction	10 (2.86)
RNA processing	10 (2.86)
Development	8 (2.29)
Organelle activity	8 (2.29)
Secondary metabolism	7 (2.00)
Hormone regulation	5 (1.43)
Abiotic stress-related	5 (1.43)
Lipid metabolism	4 (1.14)
Protein metabolism	4 (1.14)
Protein transport	4 (1.14)
Protein folding	2 (0.57)
Function not annotated	52 (14.86)
No hits found	90 (25.71)

These transcripts were pooled from clusters III, IV and V (Figure 5), with each individual transcript then allocated a functional category based on annotation and literature searches

**Table 2:** Primer sets used in Q-PCR analysis.

**Candidate**	**Affymetrix probe set**	**Acquisition temperature**	**Sense**	**Antisense**	**Product size (bp)**
			Sequence	Sequence	
ASY1	Ta.9186.1.S1_at	83	AGGACTCCCACAAGCAATCG	ACCTGCTGGAGGATCGGCTC	159
DMC1	Ta.30833.1.S1_at	80	GCTGAGGAAAGGCAAAGGCG	CGGTGCAGTAGCCTTTGTCGATTT	195
MSH4	Ta.25861.1.A1_at	83	CTGAAGGATGGTGTCCGACG	AGGTCCTGCAATGCTTCACG	252
MSH6	Ta.24096.1.S1_at	80	CATAATATTGGCACAGATTGGAG	CTGACGAAAGCACGGAAGC	161
PHS1	TaAffx.47591.1.S1_at	80	TGATGCTGCTGGTGGAAATTCG	CGGACACTAGGCATGATAGGCG	270
RAD51B	Ta.7197.1.A1_at	81	CTGCCTGGCTGAAGCTGAAG	GATAGAACAAGCAAACTATGGGAG	267
RAD51C	Ta.10540.1.S1_at	79	TATTGATACAGAGGGCAGTTTC	AGCTGCATATTCGGAAGTAG	173
RAD54	Ta.7706.1.S1_at	82	GCTGTCGCAAACCCTTTCG	GTCGTAGGCACCAATCATCCATC	344
RPA	Ta.6986.1.S1_at	83	GAATGTCTTCCGTGAACCG	CATCTAAGGACGGGTGCTAG	274
WM5	Ta.25342.1.S1_at	81	CATCGAGTGGGCTTTAGCTATAG	CAACACGTAGTACGATAGATCCAAG	178
WMC3*	Ta.28776.1.A1_at	79	CGACCGACGGAATTGATATG	CTGTGAGAAGTTTCAGCATTATC	171
WMC4	Ta.30383.1.A1_at	80	GATTCCCAAATGTTCGAC	CAAATTGTCCTCCTTACG	266
WMC5	Ta.3224.2.S1_at	81	CTTGCTGCTCCCAATGATAATTC	CAGTTAGCTGCCAGGTCATCTTTC	245
WMC6	Ta.3385.1.A1_at	80	CGGACAGTCCAGATGTGC	CTATGCTTGCTGCCTGATG	271
WMC10*	TaAffx.38162.1.S1_at	79	AGGAGTTGACATGACAAGATTAGGGAGG	GCCACTTCACATGCACCCATCTAATC	166
					
**Controls**					
Cyclophilin		82	CAAGCCGCTGCACTACAAGG	AGGGGACGGTGCAGATGAA	227
EFA1		82	CAGATTGGCAACGGCTACG	CGGACAGCAAAACGACCAAG	227
*GAPdH*		*78*	TTCAACATCATTCCAAGCAGCA	CGTAACCCAAAATGCCCTTG	220
Actin		77	GACAATGGAACCGGAATGGTC	GTGTGATGCCAGATTTTCTCCAT	236
*Flat gene 1*	*Ta.9657.1.S1_at*	*83*	CCCTCAGACCGCTGGCAA	GAGGCGGACAGACATGGAAGAA	300
*Flat gene 2*	*Ta.28350.1.S1_a_at*	*80*	ACGCAGCTACCTGTATCATTCGGATC	GAAGCGACGATGTCCACATGACC	160

Primer sets used and the product sizes obtained in addition to the acquisition temperatures used. Control genes in italics were those selected by geNorm for normalisation.

* indicates those transcripts that have an unclear orientation (not known whether sense or antisense).

**Table 3:** Q-PCR correlation of 15 candidates with one another.

	*TaASY1*	*TaDMC1*	*TaMSH4*	*TaMSH6*	*TaPHS1*	*TaRAD51B*	*TaRAD51CS*	*TaRAD54*	*TaRPA*	*TaWM5*	*TaWMC#3*	*TaWMC#4*	*TaWMC#5*	*TaWMC#6*	*TaWMC#10*
*TaASY1*	1.00														
*TaDMC1*	**0.98**	1.00													
*TaMSH4*	*0.90*	**0.95**	1.00												
*TaMSH6*	**0.99**	**0.98**	*0.92*	1.00											
*TaPHS1*	**0.97**	**0.98**	**0.97**	**0.98**	1.00										
*TaRAD51B*	*0.94*	0.87	0.76	0.89	0.88	1.00									
*TaRAD51CS*	*0.93*	**0.96**	*0.94*	**0.95**	**0.96**	0.79	1.00								
*TaRAD54*	**0.99**	**0.99**	*0.92*	**0.98**	**0.97**	*0.92*	*0.94*	1.00							
*TaRPA*	**0.99**	**0.98**	*0.93*	**0.99**	**0.99**	*0.91*	**0.95**	**0.98**	1.00						
*TaWM5*	**0.97**	**0.99**	**0.97**	**0.97**	**0.99**	0.87	**0.95**	**0.99**	**0.98**	1.00					
*TaWMC#3*	0.87	0.84	0.67	0.89	0.79	0.76	0.84	0.83	0.84	0.78	1.00				
*TaWMC#4*	**0.99**	**1.00**	**0.96**	**0.99**	**0.99**	0.89	**0.96**	**0.99**	**0.99**	**1.00**	0.82	1.00			
*TaWMC#5*	**0.97**	**0.96**	*0.94*	**0.97**	**0.99**	0.89	**0.95**	**0.95**	**0.99**	**0.96**	0.82	**0.98**	1.00		
*TaWMC#6*	*0.94*	**0.98**	**0.97**	**0.97**	**0.99**	0.80	**0.97**	*0.94*	**0.97**	**0.97**	0.81	**0.98**	**0.97**	1.00	
*TaWMC#10*	-0.74	-0.65	-0.46	-0.74	-0.62	-0.71	-0.70	-0.68	-0.69	-0.59	-0.93	-0.65	-0.68	-0.62	1

Q-PCR products with a transcript correlation greater than or equal to 0.95 and less than 1 are in bold, while correlations smaller than 0.95 but greater than or equal to 0.9 are italicized.
